# Hypercalcemia–leukocytosis syndrome from non-schistosomiasis-associated squamous cell carcinoma of the urinary bladder: a case report and review of the literature

**DOI:** 10.1186/s13256-023-03860-x

**Published:** 2023-04-12

**Authors:** Ijeoma N. C. Chibuzo, Rion Healy, Umi Hatimy, Vincent C. Tang

**Affiliations:** 1 Department of Urology, Stepping Hill Hospital, Poplar Grove, Stockport, SK27JE, UK; 2 Department of Pathology, Stepping Hill Hospital, Poplar Grove, Stockport, SK2 7JE, UK

**Keywords:** Non-schistosomiasis-associated, Squamous cell carcinoma, Paraneoplasia, Hypercalcemia, Leukocytosis

## Abstract

**Background:**

Non-schistosomiasis-associated squamous cell carcinoma of the urinary bladder is less common in the Western world. Limited information on its possible paraneoplastic syndromes exists. Leukocytosis tends to commonly be regarded by clinicians as an indication of sepsis, rather than a feature of paraneoplasia, potential surrogate marker for recurrence, and prognostic marker. Accompanying hypercalcemia may be missed entirely.

**Case presentation:**

A 66-year-old Caucasian man presented with visible painless hematuria and symptomatic hypercalcemia. Investigations revealed a squamous cell carcinoma of the urinary bladder with marked leukocytosis. Hypercalcemia and leukocytosis resolved following radical cystectomy, recurred with nodal recurrence and regressed with radiotherapeutic control. Subsequently, serum leukocyte and calcium assays were included in his follow-up protocol. His survival was 20 months by the time of the report.

**Conclusion:**

This report highlights hypercalcemia–leukocytosis syndrome as a paraneoplastic manifestation of non-schistosomiasis-associated squamous cell carcinoma to reemphasize the need for clinicians to assay for calcium in the presence of leukocytosis in such patients. Prompt identification and control of the paraneoplastic derangements, with treatment of the cancer recurrence it may connote, is advocated to provide a chance for better long-term outcomes in these patients.

## Introduction

Hypercalcemia–leukocytosis syndrome (HLS) as a paraneoplastic syndrome (PNS) of bladder cancers is rare, although it is documented in anaplastic thyroid, lung, and penile squamous cell carcinomas (SCCs) [1, 2]. Non-schistosomiasis-associated squamous cell carcinoma (NSA-SCC) accounts for 2% of all bladder cancers in the Western world [3]. We describe leukocytosis and symptomatic hypercalcemia associated with hypophosphatemia due to an NSA-SCC of the bladder.

## Case summary

**Fig. 1:**
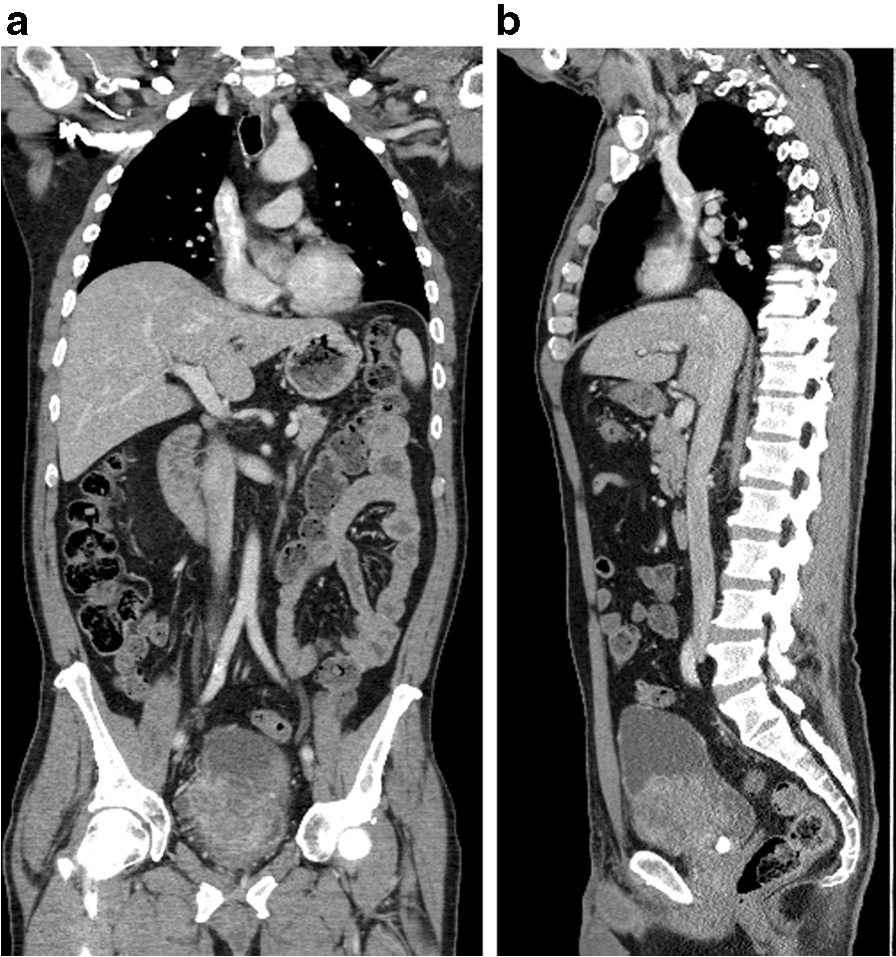
**a** Sagittal view of computed tomography scan of the thorax, abdomen, and pelvis showing the bladder tumor. **b** Coronal view of computed tomography scan of the thorax, abdomen, and pelvis showing the bladder tumor

**Fig. 2:**
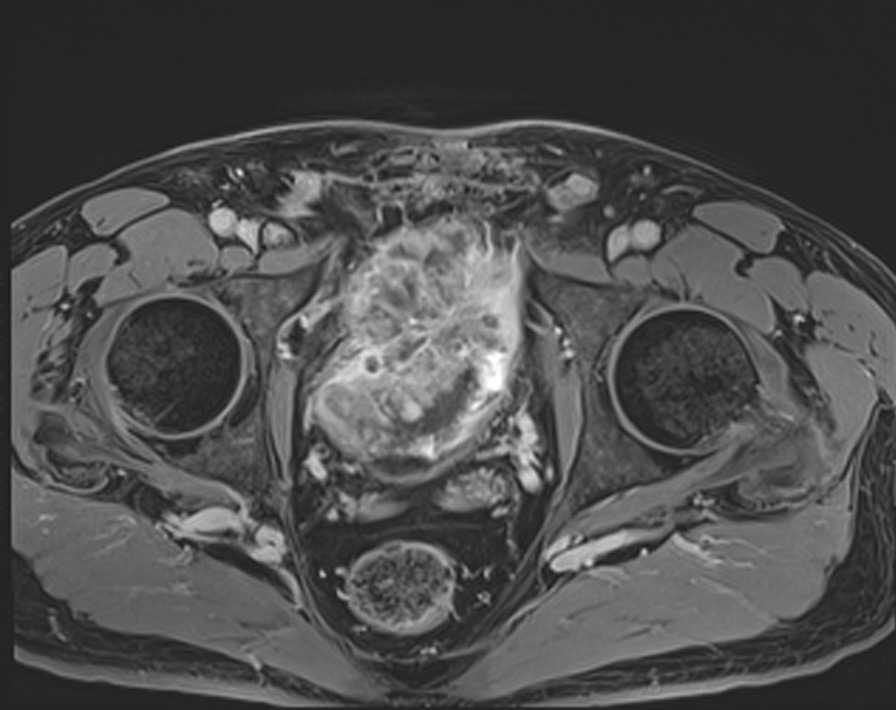
Pelvic MRI T1-weighted axial image showing anterior extrusion of the tumor on the right without pelvic side wall involvement

A 65-year-old Caucasian welder presented with intermittent visible and painless hematuria with a duration of 1 month. He was a non-smoker. He had bone pains, fatigue, and reduced mobility. His Eastern Cooperative Oncology Group (ECOG) score was 4. Flexible cystoscopy showed solid-looking tumors at the bladder base and a calculus. A computed tomography (CT) scan (Fig. 1) showed a large multifocal tumor, calculus, and extravesical stranding. There was no evidence of metastasis. He had cystolitholapaxy and transurethral resection of the bladder tumor. Histology revealed SCC. A pelvic magnetic resonance imaging (MRI) (Fig. 2) showed extravesical tumor extrusion without pelvic wall involvement.

A robot-assisted radical cystoprostatectomy, ileal conduit, and pelvic lymph node dissection was performed. Routine periodic intraoperative monitoring showed elevated ionized calcium (ical) of 1.67 mmol/L (normal: 1.15–1.30 mmol/L) at an arterial pH of 7.38 (normal: 7.4 ± 0.04). With tumor mobilization, ical increased to 1.77 mmol/L. The adjusted serum calcium level was 3.22 mmol/L (normal: 2.2–2.6 mmol/L). The serum phosphate nadir was 0.63 mmol/L (normal: 0.80–1.5 mmol/L). Given the high calcium levels, the parathyroid hormone (PTH) level was checked and found to be suppressed at 5 pg/L (normal: 18–80 pg/L), with normal procalcitonin of 0.27 ng/mL (normal: < 5 ng/mL) and alkaline phosphatase levels of 90 iU/L (normal: 20–140 iU/L). There was a rise in white cell count (WCC) titers to 41 × 10^9^/L from a preoperative value of 23.2 × 10^9^/L, with neutrophilia of 35.2 (normal: 1.7–7.5 × 10^9^/L). Antibiotics were administered. Cultures were negative. He remained apyrexial. The clinical picture was not in keeping with sepsis. Antibiotics were discontinued.

A diagnosis of a paraneoplastic syndrome causing hypercalcemia and leukocytosis was entertained. Unfortunately, PTHrP was not assayed, and a lack of facilities at ours and nearby centers precluded PTHrP and G-CSF immunotyping of the cystoprostatectomy specimen.

**Fig. 3:**
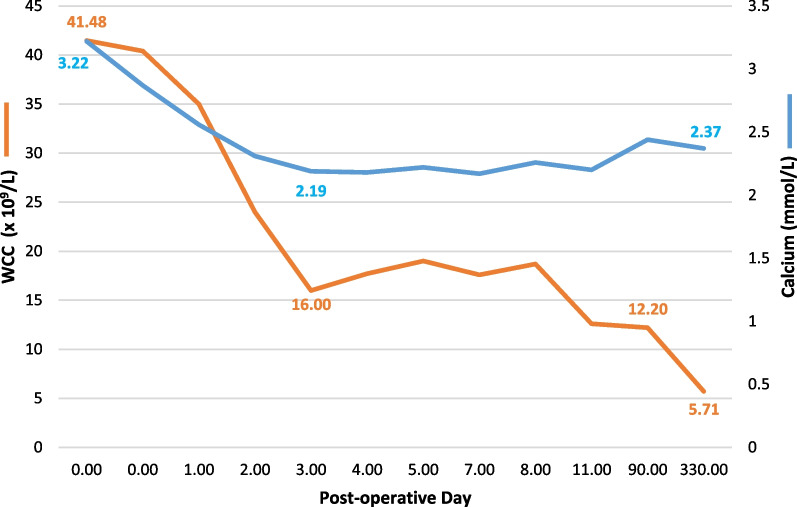
Observed trend in serum leukocyte and calcium levels

Saline boluses and phosphate supplements were administered. Serum calcium levels normalized by postoperative day 1 to 2.56 mmol/L, and steadily declined thereafter (Fig. 3). Serum phosphate normalized by day 3. Leukocytosis gradually resolved, with WCC returning to relatively normal values by the third postoperative month (Fig. 3).

**Fig. 4:**
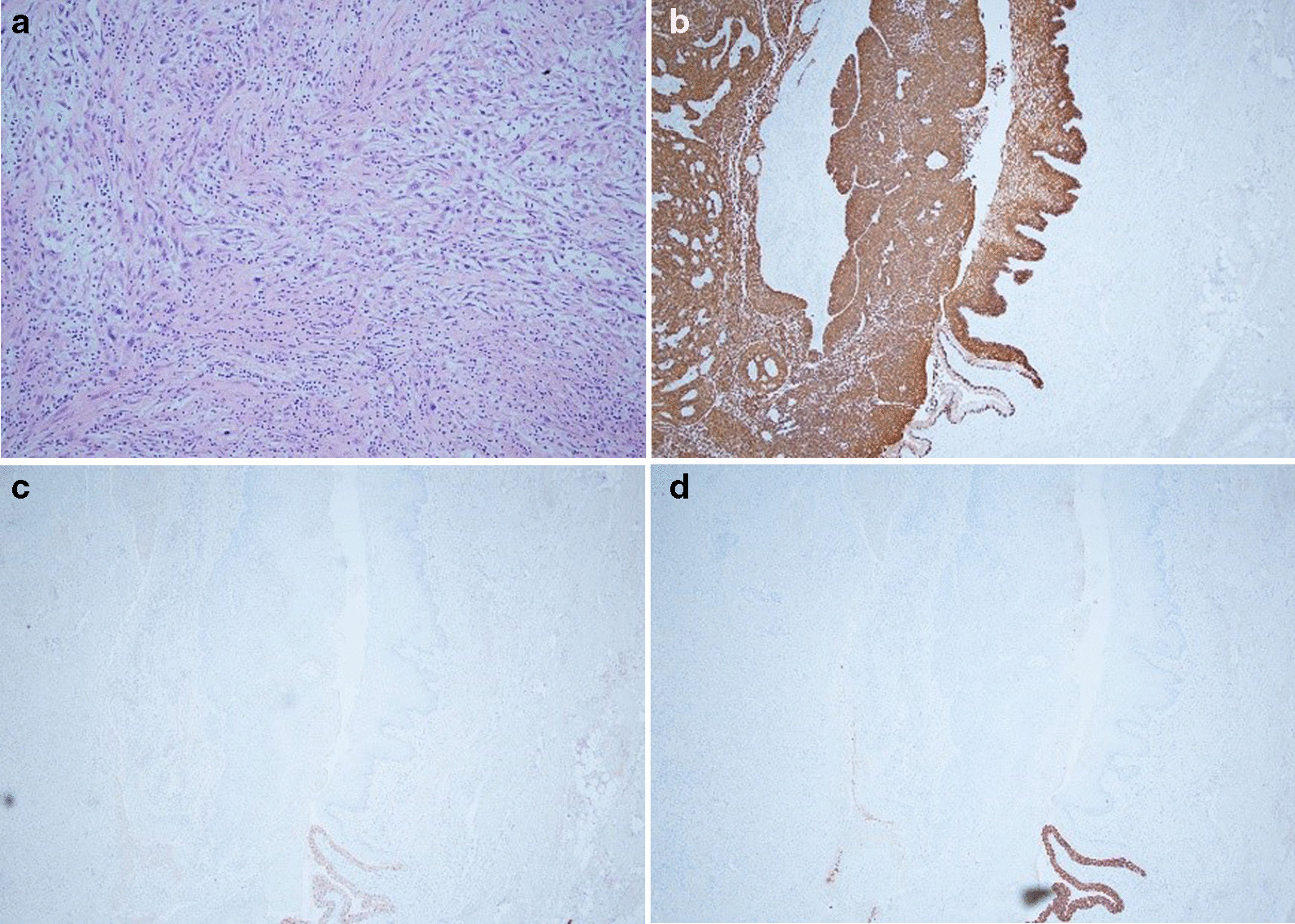
**a** Squamous cell carcinoma (SCC) with spindle cell morphology. **b** Strong SCC CK 5/6 positivity. **c** Contrast between GATA3-staining normal urothelium and GATA3-negative SCC. **d** Focal CK7 immunostaining of SCC with obvious positivity of adjacent normal urothelium

The histology showed a T3bN_1_ moderately to poorly differentiated invasive SCC with scattered areas of spindle cell morphology, lymphovascular and perineural invasion, and negative margins. It was diffusely positive for CK5/6 and p63, focally positive for CK7, and negative for CK20 and GATA 3 (Fig. 4).

Two months following discharge, he developed recurrence of malaise and bone pains and was admitted at another hospital. He had leukocytosis of 15.21 × 10^9^/L, which was treated as urosepsis. No serum calcium was assayed. WCC rose further to 18.08 × 10^9^/L. A revision CT scan revealed new retroperitoneal lymphadenopathy. He was deemed clinically unfit for chemotherapy. He received palliative radiotherapy with nodal regression on follow-up imaging. At his last review, his serum calcium and WCC were normal at 2.37 mmol/L and 5.71 × 10^9^/L, respectively. He was ECOG 1. He had a 20-month survival by the time of this report.

## Discussion

### Hypercalcemia–leukocytosis syndrome prevalence

A review of 35 bladder cancers with paraneoplastic leukocytosis in Western and Japanese literature showed nine (25.7%) with hypercalcemia and/or elevated PTHrP [4]. None of these had pure SCC. To date, there are only four other reported cases of HLS secondary to NSA-SCC of the bladder (Table 1) [5–8]. One reported pure SCC histology [6], while three were mixed transitional cell carcinoma (TCC)/SCC [5, 7, 8]. Our patient had focal CK7 staining and lacked CK20 and GATA3, emphasizing the lack of a TCC component [9]. The sarcomatoid differentiation produced P63 [10], while the squamous component produced CK5/6 positivity [9].

**Table 1:** Summary of HLS associated with NSA-SCC of the urinary bladder

References	Year	Country	Age	Sex	Surgical care	Adjuvant care	Histology	Stage	Peak WCC (× 10^9^/L)	Peak Ca (mmol/L)	GCSF (pg/mL)	PTHrP (pmol/L)	Resolution post-intervention	Survival (months from diagnosis)	Survival (months from PNS onset)
Vaidyanathan *et al*. [7]	2002	UK	NI	M	Suprapubic cystostomy and debulking; then palliative care	NI	Necrotic keratinizing moderately differentiated SCC	pT2a/b N_0_M_0_	22.2	3.28	NI	NI	No resolution after debulking	5	NI
Hirasawa *et al*. [8]	2002	Japan	51	M	Radical cystectomy and neobladder	NAC (CP)	TCC + SCC	NI	30.2	2.79	98.3	2.3	Resolved following TURBT	40*	40*
Turalic *et al*. [6]	2006	USA	51	F	Radical cystectomy and ileal conduit	NI	Poorly differentiated TCC + keratinizing SCC	pT4N_1_M_1_	34	2.97	84.9	NI	PNS from metastases following intervention	3	1
Rink *et al*. [9]	2009	Germany	53	F	Radical cystectomy and ileal conduit	AC (T)^NR^	TCC + SCC	pT3bN_1_M_0_	139.2	4.15	100	14	Resolved following cystectomy	2	NI
Index case	2020	UK	65	M	Radical cystectomy and ileal conduit	Radiotherapy	Moderately to poorly differentiated SCC with spindle cells	pT3bN_1_M_0_	41	3.22	NI	NI	Resolved following cystectomy	20*	20*

*NAC* neoadjuvant chemotherapy, *AC* adjuvant chemotherapy, *CP* cisplatin–pirarubicin (intraarterial injection), *T* paclitaxel, *NR* no response, *NI* not included, *PNS* paraneoplastic syndrome

*Follow-up duration (not survival). *Median survival for previously reported cases was 4 months*

### Paraneoplastic leukocytosis

#### Pathophysiology

**Fig. 5:**
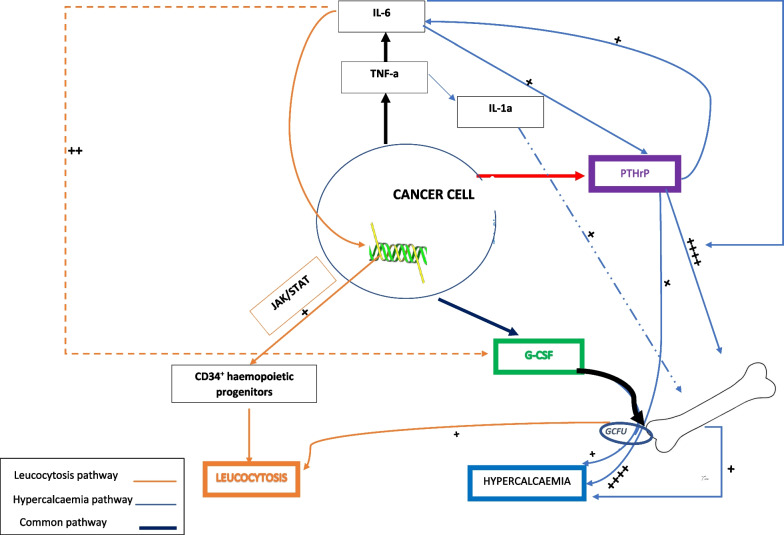
Hypercalcemia–leukocytosis syndrome (HLS) oncogenetic microsystem. *CD34*^+^ cluster of differentiation positive, *GCFU* granulocyte colony forming unit, *G-CSF* granulocyte colony stimulating factor, *IL-1a* osteoclast activating factor, *IL-6* interleukin 6, *JAK/STAT* Janus tyrosine kinase/signal transducer and activation of transcription, *PTHrP* parathyroid hormone-related peptide, *TNF-α* tumor necrosis factor-alpha

Paraneoplastic leukocytosis occurs through production of cytokines such as granulocyte colony-stimulating factor (G-CSF) more commonly, and granulocyte-macrophage colony-stimulating factor, interleukins (ILs) 1α (osteoclast-activating factor), 3, and 6, and tumor necrosis factor (TNF)-α less commonly [11]. G-CSF, produced by immature or poorly differentiated cells, stimulates bone marrow production of granulocytes [11]. TNF-α can stimulate IL-6, G-CSF, and Parathyroid hormone-related protein (PTHrP) production [12]. PTHrP activates the pro-inflammatory cytokine IL-6 and fosters G-CSF expression [2]. IL-6 is also a potent activator of signal transducer and activator of transcription 3 (STAT3) and its signalling pathways [13], which encourage proliferation in the muscle-invasive sarcomatoid SCC subtypes [14, 15]. Janus tyrosine kinase (JAK)-STAT3 signaling dysregulation promotes leukocytosis via IL-6. G-CSF and IL-6 are implicated in mild and severe hypercalcemia, respectively [16]. Thus, HLS is a product of a synergistically sustainable oncogenetic microsystem (Fig. 5) [4]. Although definite proof could not be obtained in our patient due to diagnostic challenges, the resurgence in leukocyte level elevations with cancer recurrence and normalization with disease-regression were highly suggestive.

#### Prognostic implications of paraneoplastic leukocytosis

Leukocytosis is inversely related to survival, with leukemoid reactions (> 40 × 10^9^/L) and hyperleucocytosis (> 100 × 10^9^/L) having worse prognosis. This may be because G-CSF promotes angiogenesis, invasiveness, an immunosuppressive shield around the cancer, and is produced more aggressively by metastatic foci [11]. Persistent leukocytosis post-therapy implies recurrence, poor survival, or residual tumor [6, 11]. Options for intervention include hyperhydration, hydroxyurea, allopurinol, rasburicase, urine alkalinization, and leukapheresis.

### Paraneoplastic hypercalcemia and hypophosphatemia

#### Pathophysiology

Isolated hypercalcemia is described as one of the more common paraneoplastic syndromes. Fuller Albright differentiated hypercalcemia from skeletal invasion from that due to humoral cytokine expression by malignancies, which he termed humoral hypercalcemia of malignancy (HHCM) [17]. The mechanisms for HHCM include osteolysis at sites of bone metastases in 20% of cases, tumor secretion of PTHrP, and, less commonly, an extrarenal site of calcitriol or ectopic PTH production (< 1% each) [18]. A low PTH excludes an ectopic production site. PTHrP, a PTH homolog, binds type 1 PTH receptors in bones and kidneys [12], increasing bone resorption, renal absorption of calcium, and phosphate excretion. PTHrP is a distinctive surrogate marker of SCC [19].

#### Prognosis

Hypercalcemia manifests with lethargy, gastrointestinal symptoms, bone and abdominal pains, and increased mortality, neurologic, renal, and/or cardiac impairment in severe cases [20]. Conservative care includes hydration, loop diuretics, bisphosphonates [6], denosumab, mithracin, hemodialysis, calcitonin, and glucocorticoids [21]. Persistent hypercalcemia after extirpative surgery and/or adjuvant therapy for predominant SCC is a marker of recurrence [20].

## Conclusion

To the best of our knowledge, we present the second case of HLS PNS due to predominant NSA-SCC histology, and the first with survival greater than 5 months. Although uncommon, PNS in NSA-SCC should be considered in patients presenting with unexplained leukocytosis and hypercalcemia. We recommend serum calcium assay inclusion in the evaluation of leukocytosis in patients with NSA-SCC, and follow-up measurements of both to monitor for tumor recurrence.

## Data Availability

All data generated or analyzed during this study are included in this published article.
